# Surgical management of metastatic lesions of proximal femur and the hip

**DOI:** 10.1016/j.amsu.2018.09.042

**Published:** 2018-11-02

**Authors:** Mujahid Jamil Khattak, Umair Ashraf, Zohaib Nawaz, Shahryar Noordin, Masood Umer

**Affiliations:** Aga Khan University, Karachi, Pakistan

**Keywords:** Femur, Hip, Metastatic

## Abstract

Metastatic bony lesions involving proximal femur and hip joint pose a challenge to orthopedic surgeons. Lesions in this important weight-bearing zone of the femur weaken its ability to sustain load causing pain and impending pathologic fracture. These Patients warrant multidisciplinary approach including orthopedic surgeons, oncologist and medical specialties. Management of these lesions has evolved over the last 60 years from benign neglect to internal fixation and recently to prosthetic reconstruction for optimum function. Decision for surgical approach requires consideration for location of the lesion, presence of a fracture, tumor type, cortical destruction, patient's life expectancy, patient preferences and the expected outcome. We aim to present a narrative review of the options and results of surgical management of these lesions in the light of literature.

## Highlights

• Surgical management of hip metastasis.
• Metastatic lesions of the proximal femur.
• Endoprosthetic reconstruction of hip metastasis.

## 1 Introduction

Metastatic lesions of the proximal femur and hip joint are common and present with multiple management issues. Management involves general care physicians, medical oncologists and reconstructive hip surgeons. These lesions frequently arise from breast, prostate, lung, renal, and thyroid carcinomas [[1], [2], [3]]. The tumor that metastasizes to the hip with the greatest frequency is carcinoma of the breast [[4], [5], [6]] Pathologic lesions in this important weight-bearing zone weaken its ability to sustain load causing pain and impending pathologic fracture. It is estimated that 40% of patients with pathologic fractures survive for at least 6 months after their fracture, and 30% survive for more than 1 year [7,8]. Management of these lesions varies from benign neglect to internal fixation and recently to prosthetic reconstruction for optimum function. This article aim to focus on metastatic tumors of the hip and proximal femur and present a narrative review of surgical management options of this unique clinical entity.

## 2 Historical perspective

The management of metastatic hip lesions has evolved considerably over the past 60 years. Use of light traction was the most primitive form of treatment. Coley and Higinbotham [11] initially reported that pathologic fractures could be prevented by the use of calliper splints to decrease the load on the affected bone. Their emphasis then shifted to identifying and treating symptomatic lesions before fracture. In 1950, tumor resection and replacement with bulk allograft was reported with favourable outcome for tumors involving the upper extremity [12]. Internal fixation of impending or actual fractures also became popular at the same time. However, failed internal fixation of pathologic femoral neck fractures led Francis et al., [13] in 1962, to advocate resection of the femoral head and neck as a primary treatment for lesions involving those structures. They believed that resection would provide rapid pain relief and quicker rehabilitation and it will help reducing surgical complications. They reported good outcome of their 19 patients in the series. In 1976, Harrington started advocating the use of polymethyl methacrylate (PMMA) cement as an adjunct to internal fixation in patients with bone loss in metastatic disease. In a series of 375 patients, Harrington et al. [14] excised the lesion and then performed internal fixation or prosthetic replacement and reinforcement with PMMA. He reported 94% ambulation rate in his series.

In 1980, Lane et al. [15] reported endoprosthetic replacement for pathologic lesions of the hip. Impending fracture and a life expectancy of more than 1 month was considered adequate indication for surgical intervention. Good to excellent results were obtained in all of the patients treated with either an Austin- Moore Hemiarthroplasty or a total hip replacement. In 1981, Harrington [16] reported on the use of total hip prostheses in patients with acetabular lesions. Harrington designed a larger acetabular component that would distribute the mechanical load to areas of less involved bone. Current management of these lesions focuses on thorough assessment and surgical intervention leading to quick rehabilitation and successful long-term reconstructive strategy.

## 3 Evaluation and planning

Metastatic lesion of the Hip and the femur are seen in a variety of situations. Presentation can vary from patients presenting with Hip pain in the outpatient clinic, as a specialist referral to Hip surgeon or as pathological fracture to the emergency on call orthopedic surgeon.

A thorough, multi-disciplinary pre-operative evaluation is indicated to avoid decision-making errors. Complete history and physical examination, appropriate serology, skeletal survey, bones scan, and computed tomography of the chest, abdomen, and pelvis will identify the vast majority of primary tumors for a suspected metastatic lesion. The investigation should include a medical oncologist, internist, and potentially a radiation oncologist. Biopsy may be indicated if a primary source cannot be identified [17,18]. A full discussion of the evaluation and treatment of a primary bone malignancy or unknown primary is beyond the scope of this review. For clarity, we will discuss bony lesions around the hip and will focus on the surgical decision-making centered on internal fixation or arthroplasty.

Pre-operative medical optimization is always recommended, as these patients usually present with generalized weakness and metabolic issues including, hypercalcemia or coagulopathy [19]. It is important for surgeon and the patient to consider remaining life expectancy and other medical issues before any operative intervention. Involvement of medical oncologist helps to establish the prognosis of the disease and possible role of chemotherapy or radiotherapy can be considered for better disease control and to optimize time for surgical intervention.

Patients presenting with impending pathological fracture warrant quicker assessment and early intervention [17,18]. Various methods of predicting the risk of fracture have been proposed. The Mirel's criterion is most commonly used (Table 1). The score is based on nature of the lesion, anatomic location, size, and assessment of functional pain [20]. The higher the score, greater is the risk of fracture. For example, lesions with scores of eight or higher would be treated with internal fixation (a lytic, painful sub trochanteric lesion), while lesions with lower scores (a painful, small lesion in the shaft of the radius) would be considered candidates for radiation treatment [21]. Obviously, lower extremity lesions with functional pain have a high rate of fracture and should probably be treated with prophylactic internal fixation.

**Table 1:** Mirel's Criteria for evaluating fracture risk for metastatic lesions.

Score			
Variable	1	2	3
Site	Upper Limb	Lower Limb	Peri-trochanteric
Pain	Mild	Moderate	Activity
Activity/Nature	Blastic	Mixed	Lytic
Size	1/3	1/3–2/3	>2/3rd

Patients with a single Hip lesion and an unknown known primary tumor need to be evaluated with caution. Primary bone tumors, such as chondrosarcoma, typically present in the hip area and it is not uncommon for patients to undergo hip replacement or insertion of an intramedullary device and the pathologist subsequently reporting a primary bone sarcoma [22].

Preoperative embolization is recommended before surgery in cases of metastatic renal and thyroid cancer [9,10]. These tumors are highly vascular, and hemorrhage can complicate surgery. Embolization should occur as close to the time of surgery as possible. Waiting longer than twenty-four hours between embolization and surgery may produce fevers, rigors, and diaphoresis related to tumor necrosis.

## 4 Current techniques

**Fig. 1:**
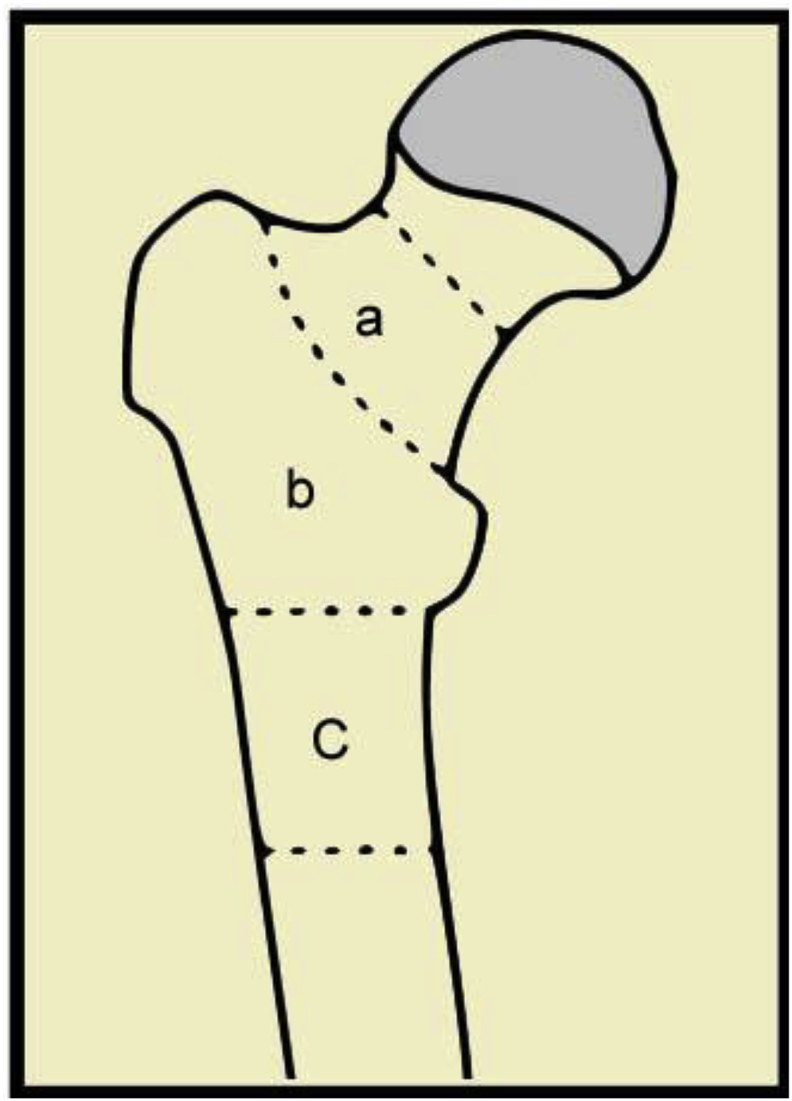
Illustration of the proximal femur demonstrating the anatomic areas for consideration of surgical intervention. Area “a” consists of the isthmus to the base of the femoral neck, “b” is the trochanteric region, and “c” is the sub trochanteric region to 5 cm below the lesser trochanter. For lesions spanning multiple areas, the area most affected by the lesion is primarily important for surgical planning.

Surgical intervention for metastatic hip lesions helps to obtain biopsy for diagnosis, alleviate pain and restore function. Ideally, these patients should have one-time appropriately planned operative intervention for optimum pain relief and quick functional rehabilitation. Selection of surgical technique varies from open reduction and fixation or intramedullary nailing of the pathologic fractures to prosthetic replacement of the diseased proximal femur and the Hip. Decision for surgical approach requires consideration for location of the lesion (Fig. 1), presence of a fracture, tumor type, cortical destruction, patient's life expectancy, patient preferences, and the expected outcome [[17], [18], [19], [20]]. Only a few studies have rigorously compared outcomes among implant types [17,18]. A survey study of 98 orthopedic oncologists demonstrated large variation in the physicians' preferred surgical strategies emphasizing the need for further study to improve understanding of surgical outcomes [21].

Although preferences vary among surgeons, in general, a pathologic fracture is treated surgically when the patient was expected to live longer than 30 days. An impending fracture is also generally treated surgically when the patient is expected to live longer than 30 days and has substantial bone destruction or pain on load bearing. Obviously, these lesions are best treated in the “impending-fracture” stage as there is less functional loss and rehabilitation is quicker if the bone has not broken [22,23].

### 4.1 Osteosynthesis

**Fig. 2:**
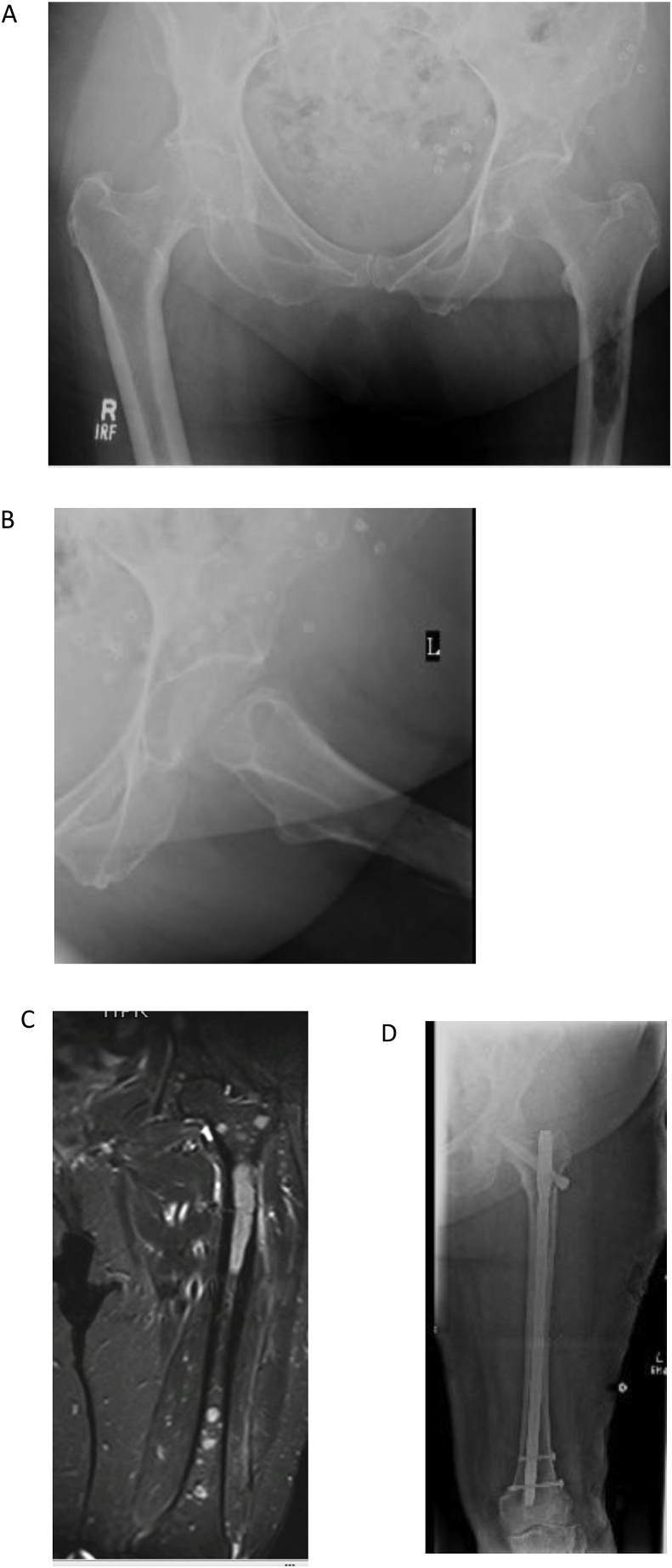
79 year-old, female with lytic lesion in left proximal femur. A: AP view showing the lesion in prximal. part of left Femur. B: lateral view of the left Hip. C: MRI of the left Femur showing the extent of the lesion and its confinement to bone. D: left femur IM nailing was performed, histopathology confirmed Plasma Cell tumor, at 2 years follow up, patient is fully ambulant with no pain or residual disease.

Patients with trochanteric or sub trochanteric lesions with preserved head and neck of femur are amenable to stabilization with intramedullary nails, plates or dynamic Hip screw fixation device. Intramedullary nailing is primarily used in patients with limited bone loss and trochanteric or sub-trochanteric lesions. While aiming to stabilize the whole length of the femur with intramedullary nail, screw is also passed through the nail into the head and neck of the femur to minimise chances of future collapse and further surgical intervention (Fig. 2). Open reduction and internal fixation (ORIF) is typically used in patients with small focal lesions around the trochanteric area [24] Pathological fractures or lesions are however, less likely to heal and reoperation rate is therefore higher in this group of patients. Fracture fixation or Stabilization is therefore preferred over prosthetic replacement in patients with limited function or life expectancy of less then three months [25]. Surgery in these cases is a palliative measure to relieve pain, facilitate nursing care and is performed with or without metastatic resection and bone cement augmentation for better stabilization and quicker rehabilitation.

Osteosynthesis in metastatic lesions has been reported extensively. Piccioli et al. [26] studied functional outcomes in a group of 80 patients with the proximal femur pathologic fractures treated with a titanium proximal nail. All patients in the study reported pain relief and improvement in the quality of life. However, based on the long term follow up, authors concluded that intramedullary nail fixation may fail requiring further surgical intervention and the procedure should be reserved for patients with limited life expectancy. Steensma et al. [27] reported implant exchange after failure with 6.1% of intramedullary nailing procedures (5 of 82) and 42% of open reduction and internal fixation procedures. Wedin and Bauer [28] demonstrated 13% failure after intramedullary nailing and 25% after the use of a dynamic hip screw for reduction and fixation of lesions in the proximal femur.

Osteosynthesis procedures are technically easier and can be performed by general orthopedic surgeons. The other advantages include less anaesthetic time and blood loss and can be performed in selected group of patients with careful consideration of the location of the lesion and overall medical condition and life expectancy of these patients.

### 4.2 Prosthetic reconstruction

Proximal femur resection and prosthetic reconstruction is preferred in patients with extensive bone destruction, in patients with pathologic fractures, in tumors resistant to radiation therapy and in patients with more proximal metastatic lesions (Fig. 1). Use of custom and modular proximal femoral endoprosthetic replacements in metastatic lesions have been reported extensively [[25], [26], [27], [28], [29]]. It allows quicker rehabilitation and minimise chances of further tumor-related problems such as non-union and implants failures or exchange [[29], [30], [31], [32]].

**Fig. 3:**
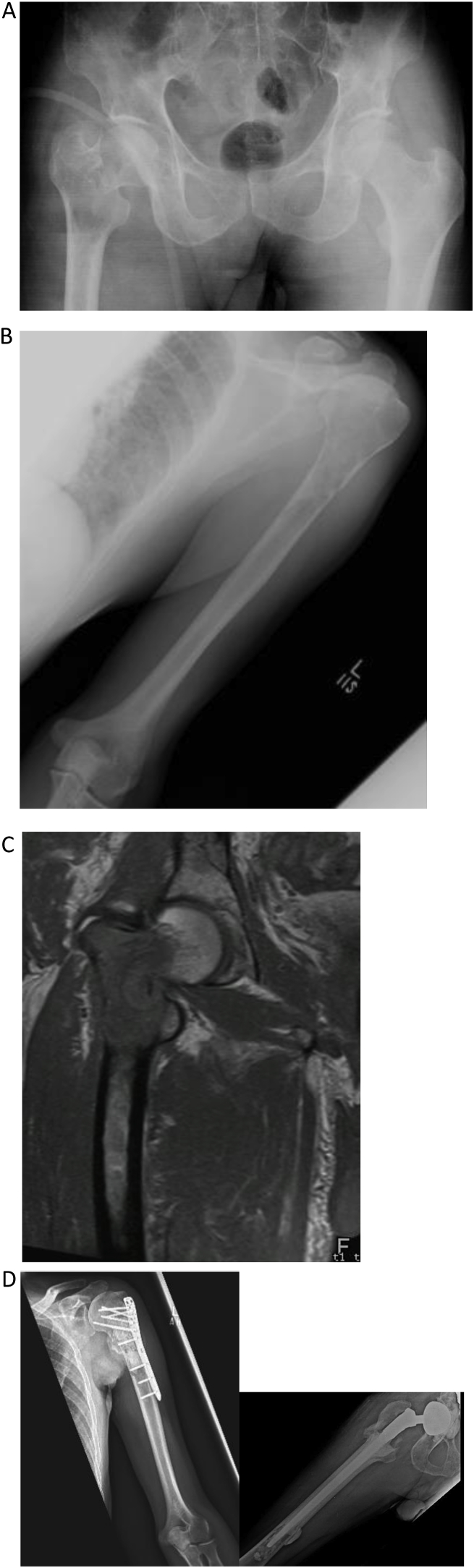
45-year-old male, known to have multiple myeloma, presented with pain in left shoulder and right hip. A: x-ray Pelvis showing pathological fracture right Femur. B: X-ray left Humerus with lytic lesion. C: MRI showing fracture and the extent of the lesion in the proximal Femur. D: ORIF left proximal Humerus with cement and right-sided proximal femur replacement with THR with dual mobility acetabular cup was performed for complete pain relief and function.

Lesions of the femoral head and neck region without acetabular involvement can be treated by Hemiarthroplasty (Fig. 3). This procedure is technically easier to perform, has less chance of hip dislocation because of large femoral head size as and can provide a satisfactory short-term functional outcome. Hemiarthroplasty however, is associated with residual hip and thigh pain because of aseptic loosening and acetabular wear. Although bipolar implants are now more commonly used, it is still a challenge and Hemiarthroplasty eventually requires revision to total hip replacement [[33], [34], [35]]. This procedure is still a good option for some patients with guarded prognosis and short life expectancy.

**Fig. 4:**
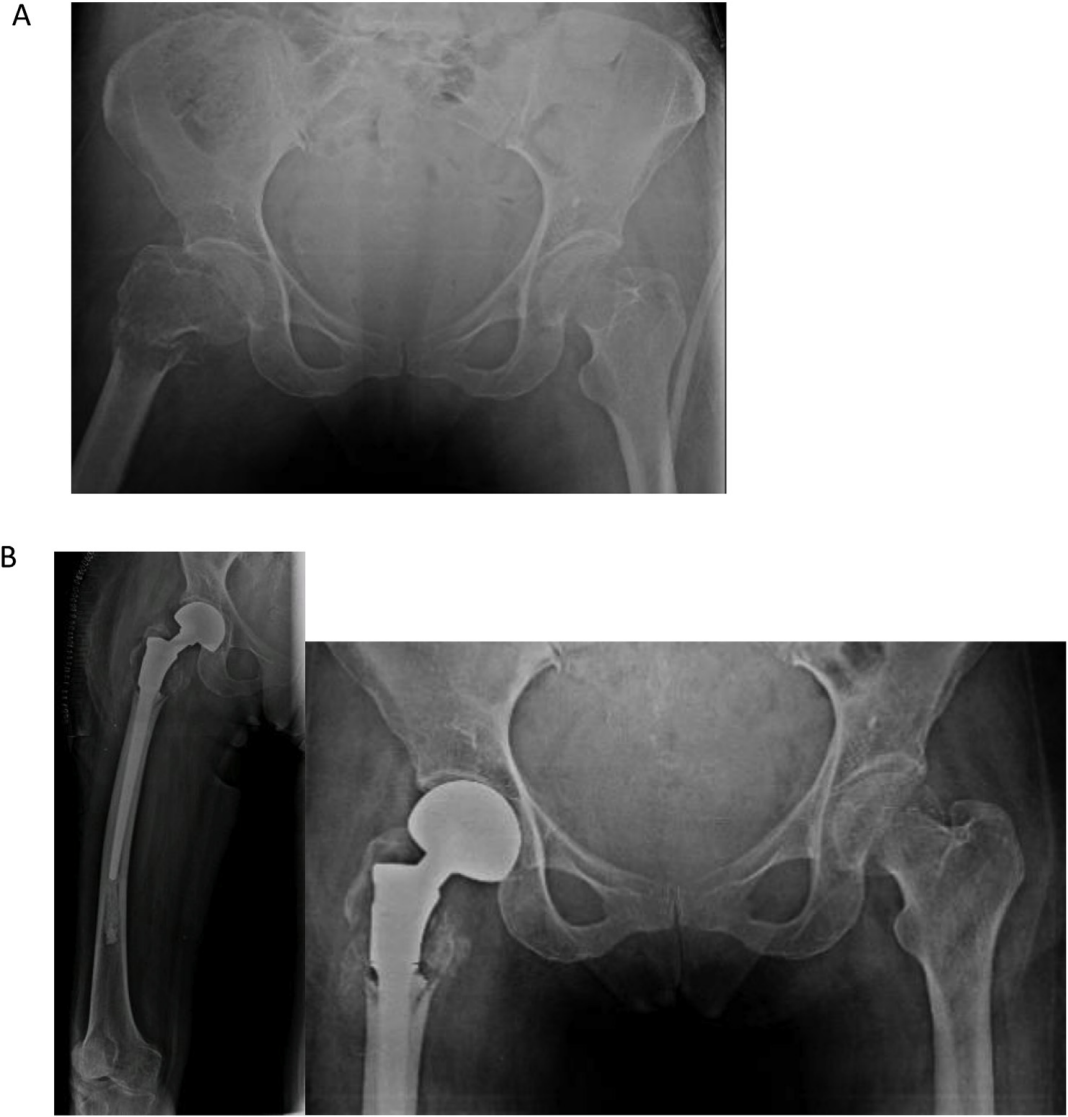
55 years old female known case of metastatic Carcinoma endometrium with metastases to lungs, bones and peritoneum presented with right leg pain. A: X-ray Pelvis showing lytic lesion in the proximal Femur with pathological fracture. B:Palliative surgery was performed for pain relief and nursing care, right sided Proximal Femur Replacement with Bipolar hemiarthroplasty.

In comparison to Hemiarthroplasty, total hip replacement eliminates chances of loosening, acetabular wear, residual hip and thigh pain and has better long-term results. The availability and use of larger femoral heads has substantially reduced the rate of dislocation in total hip replacements [36,37]. Hip surgeons therefore prefer to perform Total Hip replacement as single stage procedure in patients with or without acetabular involvement [[38], [39], [40]]. The availability of modern modular systems provides choices of femoral head sizes, calcar replacement femoral stems with trochanteric reattachments, variable sizes of femoral component shaft, length and adjustment of femoral anteversion. There are also options of using polished (cemented) or a hydroxyapatite-coated collar at the bone-prosthesis junction for osseous integration. Tumor resection in these patients can be carried out following oncological principles with wide margin resection for primary tumors and palliative reconstruction avoiding extra-articular resection for metastatic lesions. After appropriate resection, total hip replacement is performed and the acetabulum and proximal femur is reconstructed based on the amount of bony destruction on either side (Fig. 4). Proximal femur replacement prosthesis is readily available and in patients with spared greater trochanter it is subsequently reattached to the endoprosthesis with the trochanteric reattachment plate and screws or cable-grip wires. If trochanteric sparing is not possible, the abductor mechanism is sutured to vastus lateralis and the fascia lata [40].

Prosthetic replacement appears better then stabilization or fixation as it allows immediate weight bearing, quick rehabilitation and superior long-term results. Comparing the outcome of prosthetic reconstruction with intramedullary nailing and open reduction and fixation for proximal femur fractures in metastatic lesions of proximal Femur, Stein et al. reported that prosthetic reconstruction was most durable over time with no failures. Although blood loss and anesthesia time was reported higher for prosthetic reconstruction as compared to intramedullary nailing or ORIF, there was no difference in 30-day systemic complication rates among three surgical strategies. Prosthetic reconstruction however was associated with higher rate of infection [41] and this has also been reported in other studies [42,43] Dislocation is another complication of prosthetic replacement, with rates varying from 1.7% to 20% [42,43]. High dislocation rate can be attributed to the destruction and resection of soft tissues around the hip, including the muscles and hip capsule in most cases. Availability and use of large size femoral heads seems to reduce the risk of dislocation. Recent introduction of dual mobility (dual articulation acetabular component) in total hip replacement is a useful addition in hip surgeons armamentarium. Dual mobility hip replacements has substantially reduced dislocation rate in primary and revision total hip replacements and its use in tumor related hip surgery is getting increasingly popular [44].

## 5 Conclusion

Surgical management of metastatic lesions of the Hip is complex and major operative intervention. Location and extent of the lesion should be carefully assessed by advanced imaging techniques. Identification of primary tumor and Patient's medical condition needs careful consideration. Objective assessment of patient's comorbidities and cancer status can be done by the available scoring systems [[45], [46], [47]]. Surgical intervention must be planned in coordination with medical oncologists and physicians for comprehensive perioperative management. Experienced oncological and hip surgeons can perform endoprosthetic reconstruction around the hip in selected patients allowing quicker rehabilitation. This reconstruction method is durable but with inherent risks of infection and dislocation. Introduction of computer based navigation and patient specific instrumentation has the potential to increase accuracy of tumor resection and decrease the incidence of implant-specific complications [[48], [49], [50]]. Poor cancer status, timing, estimated life expectancy and available surgical expertise should be considered in the choice of surgical strategy to optimize the outcome of surgery in these patients.

## Provenance and peer review

Not commissioned, externally peer reviewed.

## Conflicts of interest

None.

## Sources of funding

None.

## Ethical approval

Not required.

## Trial registry number – ISRCTN

NA.

## Author contribution

Mujahid Jamil: first and corresponding author, idea, manuscript writing and editing.

All others: reviewing and suggesstions, editing.

## Guarantor

Mujahid Jamil.
